# A relationship between slide quality and image quality in whole slide imaging (WSI)

**DOI:** 10.1186/1746-1596-3-S1-S12

**Published:** 2008-07-15

**Authors:** Yukako Yagi, John R Gilbertson

**Affiliations:** 1 Department of Pathology, Harvard Medical School, Boston, USA

## Abstract

This study examined the effect of tissue section thickness and consistency – parameters outside the direct control of the imaging devices themselves – on WSI capture speed and image quality. Preliminary data indicates that thinner, more consistent tissue sectioning (such as those produced by automated tissue sectioning robots) result in significantly faster WSI capture times and better image quality.

A variety of tissue types (including human breast, mouse embryo, mouse brain, etc.) were sectioned using an (AS-200) Automated Tissue Sectioning System (Kurabo Industries, Osaka Japan) at thicknesses from 2 – 9 μm (at one μm intervals) and stained with H&E by a standard method. The resulting slides were imaged with 5 different WSI devices (ScanScope CS, Aperio, CA, iScan, BioImagene, CA, DX40, DMetrix, AZ, NanoZoomer, Hamamatsu Photonics K.K., Japan, Mirax Scan, Carl Zeiss Inc., Germany) with sampling periods of 0.43 – 0.69 μm/pixel. Slides with different tissue thicknesses were compared for image quality, appropriate number of focus points, and overall scanning speed.

Thinner sections (ie 3 μm sections versus 7 μm) required significantly fewer focus points and had significantly lower (10–15%) capture times. Improvement was seen with all devices and tissues tested. Furthermore, a panel of experienced pathologist judged image quality to be significantly better (for example, with better apparent resolution of nucleoli) with the thinner sections.

Automated tissue sectioning is a very new technology; however, the AS-200 seems to be able to produce thinner, more consistent, flatter sections than manual methods at reasonably high throughput. The resulting tissue sections seem to be easier for a WSI system's focusing systems to deal with (compared to manually cut slides). Teaming an automated tissue-sectioning device with a WSI device shows promise in producing faster WSI throughput with better image quality.

## Background

Technologies in WSI have been improving for the past last decade [1]. A variety of scanners are available, with faster scanning speed and higher image quality [2]. However, there are still some issues we must solve before implementation in the clinical environment such as the system stability, consistency of image quality, etc. Also during our clinical trial period, we found that slide quality, compression, focusing and so on influence image quality. At user side, it is not easy to improve focus algorithm and compression algorithm except compression ratio. The other hand, we could improve the slide quality at our end. Major slide problems are wrinkle, thickness, bubble, and variable thickness across the tissue section. Therefore, we have studied the relationship between slide quality and image quality using six different scanners and different uniform thickness of histology slides to see if this improves image quality and other measures effectiveness such as scanning time and the number of focus points (the number of points on the slide that the system uses for auto focus).

## Methods

A variety of tissue types (including human breast, mouse embryo, mouse brain.) were sectioned using an (AS-200) Automated Tissue Sectioning System (Kurabo Industries, Osaka Japan) at thicknesses from 2 – 9 μm (at 1–2 μm intervals) and stained with H&E using standard methods at the Massachusetts General Hospital. The resulting slides were imaged with 6 different WSI devices (ScanScope CS, Aperio, CA, iScan, BioImagene, CA, DX40, DMetrix, AZ, NanoZoomer, Hamamatsu Photonics K.K., Japan, Mirax Scan, Carl Zeiss Inc., Germany) with sampling periods of 0.43 – 0.69 μm/pixel (depending on instrument). Slides with different tissue thicknesses were compared for image quality, appropriate number of focus points, and overall scanning speed. Some devices do not allow users to change locations and/or number of focus points. Those devices were compared only image quality and scanning time.

## Results

Thinner sections (i.e. 3 μm sections versus 7 μm) required significantly fewer focus points and had significantly lower (10–15%) capture times. Improvement was seen with all devices and tissues tested. Furthermore, a panel of experienced pathologist judged image quality to be significantly better (for example, with better apparent resolution of nucleoli) with the thinner sections.

### Focus points

Two devices allowed us to change the location and number of focus points.

10 × 10 cm tissue sections cut in consistent 2 μm tissue section was scanned by automated mode. 36 and 40 points were selected by automated mode. We compared image quality in the automated mode to that found with manual selection of 1, 3, and 6 focus points. Both systems showed equivalent quality between 6 manual focus points and automated mode. Pathologists, engineers and in-house image evaluation software evaluated image quality. By reducing focus points, scanning time was reduced 10–15%.

### Scanning speed

5 out of 6 scanners scanned 2 μm sections fastest with automated mode. However 9 μm sections were not slowest with most scanners. The difference between 2 μm and slowest slide was average 2 min.

### Image quality

A panel of experienced pathologist judged image quality to be significantly better (for example, with better apparent resolution of nucleoli) with the thinner sections.

**Figure 1:**
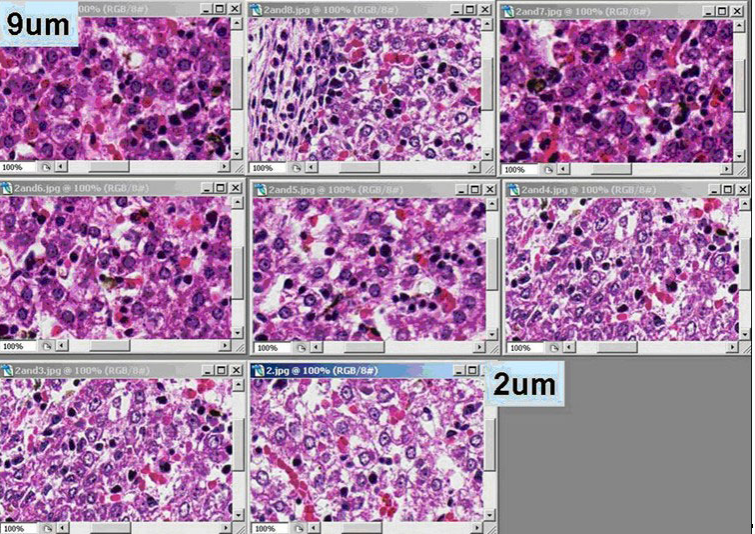
**Examples of images made on the same images but of different tissues**. Examples of images made on the same images but of tissues sections cut a various thickness (2 μm – 9 μm).

Figure 1 shows the difference between 2 μm–9 μm. 2 μm image shows more detail than 9 μm.

**Figure 2:**
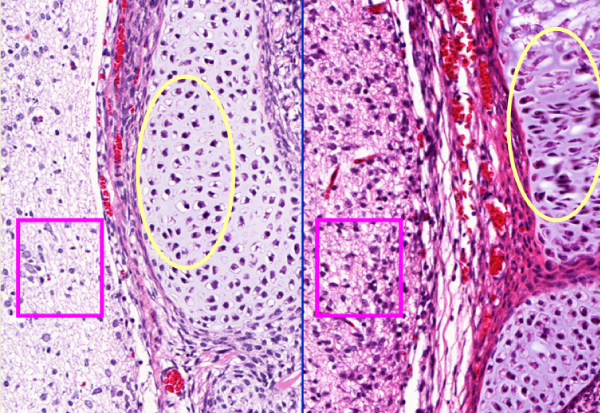
**Images from the same device and specimen but different tissue thickness**. Images from the same device and specimen but different tissue thickness (left 2 μm and right 9 μm). Note the differences in image quality and focus.

Figure 2 shows the detail of difference between 2 μm and 9 μm. In Figure 2, the 9 μm section (right) shows areas out of focus in the cartilage but the 2 μm section (left) is in focus and shows clear detail (circle line in Figure 2).

## Conclusion

Automated tissue sectioning is a very new technology; however, the AS-200 seems to be able to produce thinner, more consistent, flatter sections than manual methods at reasonably high throughput. The resulting tissue sections seem to be easier for a WSI system's focusing systems to deal with (compared to manually cut slides). Teaming an automated tissue-sectioning device with a WSI device shows promise in producing faster WSI throughput with better image quality. The automated tissue-sectioning machine is not available in US or European markets yet. However, it appears the automated histology devices, including automated tissue sectioning devices, seem to provide enhancement of image quality in WSI systems. We will keep testing and designing devices for the implementation of WSI in clinical environment and the automated histology lab of the future.

## References

[B1] Gilbertson JR, Patel AA, Yagi Y, Gu J, Ogilvie RW (2005). Clinical slide digitization – whole slide imaging in clinical practice [chapter]. Virtual microscopy and virtual slides in teaching, diagnosis and research.

[B2] Rojo MG, García GB, Mateos CP, García JG, Vicente MC (2006). Critical comparison of 31 commercially available digital slide systems in pathology. Int J Surg Pathol.

